# Dosimetric benefits of respiratory gating: a preliminary study

**DOI:** 10.1120/jacmp.v5i1.1990

**Published:** 2004-05-25

**Authors:** Laura E. Butler, Kenneth M. Forster, Craig W. Stevens, Charles Bloch, H. Helen Liu, Susan L. Tucker, Ritsuko Komaki, Zhongxing Liao, George Starkschall

**Affiliations:** ^1^ Department of Radiation Physics The University of Texas M.D. Anderson Cancer Center Houston Texas 77030; ^2^ Department of Radiation Oncology The University of Texas M.D. Anderson Cancer Center Houston Texas 77030; ^3^ Department of Biomathematics The University of Texas M.D. Anderson Cancer Center Houston Texas 77030; ^4^ Program in Medical Physics The University of Texas Graduate School of Biomedical Sciences at Houston Houston TX 77030

**Keywords:** respiratory gating, treatment planning

## Abstract

In this study, we compared the amount of lung tissue irradiated when respiratory gating was imposed during expiration with the amount of lung tissue irradiated when gating was imposed during inspiration. Our hypothesis was that the amount of lung tissue spared increased as inspiration increased. Computed tomography (CT) image data sets were acquired for 10 patients diagnosed with primary bronchogenic carcinoma. Data sets were acquired during free breathing, during breath‐holds at 0% tidal volume and 100% tidal volume, and, when possible, at deep inspiration corresponding to approximately 60% vital capacity. Two treatment plans were developed on the basis of each of the gated data sets: one in which the treatment portals were those of the free‐breathing plan, and the other in which the treatment portals were based on the gated planning target volumes. Dose‐mass histograms of the lungs calculated at 0% tidal volume were compared to those calculated at deep inspiration and at 100% tidal volume. Data extracted from the dose‐mass histograms were used to determine the most dosimetrically beneficial point to gate, the reduction in the amount of irradiated lung tissue that resulted from gating, and any disease characteristics that might predict a greater need for gating. The data showed a reduction in the mass of normal tissue irradiated when treatment portals based on the gated planning target volume were used. More normal lung tissue was spared at deep inspiration than at the other two gating points for all patients, but normal lung tissue was spared at every point in the respiratory cycle. No significant differences in the amount of irradiated tissue by disease characteristic were identified. Respiratory gating of thoracic radiation treatments can often improve the quality of the treatment plan, but it may not be possible to determine which patients may benefit from gating prior to performing the actual treatment planning.

PACS numbers 87.53 –j; 87.53.Tf

## I. INTRODUCTION

Several authors have quantified respiration‐induced organ motion. In 1990, Ross et al.^(1)^ reported movement of thoracic lesions of around 1.0 cm that resulted from respiration and cardiac motion. Korin et al.^(2)^ reported movements of the upper abdominal organs of 1.3 cm during normal respiration and 3.9 cm during deep respiration. Davies et al.^(3)^ also reported movement of the liver and diaphragm of 0.7 to 2.8 cm during normal respiration. Kubo and Hill^(4)^ studied the magnitude of respiration‐induced organ motion using fluoroscopic images. The diaphragm was found to move 1.5 to 2 cm in the superior‐inferior direction. The chest wall was reported to move laterally by 0.5 to 0.7 cm.

To account for such organ motion, the International Commission on Radiation Units and Measurements (ICRU) has defined an internal target volume (ITV) as including a margin around the clinical target volume (CTV) that explicitly accounts for organ motion.^(5)^ ^,^ ^(6)^ This margin, when properly established, allows adequate treatment of the tumor, but it may increase the amount of normal tissue that is irradiated to a high dose.^(7)^ Reducing the ITV margin could potentially improve the quality of radiation treatment by reducing the amount of normal lung tissue that is irradiated.

Several approaches have been studied to reduce the ITV margin for respiratory motion. One approach involves directing the patient in a voluntary deep inspiration breath‐hold (DIBH). Hanley et al.^(8)^ studied the dosimetric effects of a DIBH technique. They advocated this technique because deep inspiration allows a reduction in lung density while the breath‐hold immobilized the tumor. Consequently, not only can smaller margins be used to decrease the amount of normal tissue irradiated, but the fractional volume of irradiated lung tissue also decreases, because of the decrease in lung density resulting from the DIBH maneuver. The second approach to reduce the ITV margin temporarily immobilizes the patient's respiration using active breathing control (ABC), an approach first described by Wong et al,^(9)^ who monitored patients' respiratory cycles with a spirometer, temporarily blocking airflow at a predetermined lung volume. The duration of the breath‐hold is determined on the basis of patient comfort. Radiation is delivered only during the breath‐hold period. Wong et al. indicated that the ABC technique accurately immobilized the tumor in a reproducible position while reducing the lung density. A third approach involves gating the delivery of radiation while the patient is breathing freely. Ohara et al.^(10)^ studied the approach of gating the linear accelerator to deliver radiation at the end of expiration. They selected end expiration for the gating because they believed that at this point in the respiratory cycle, the diaphragm was fully relaxed and the position was its most reproducible.

In each of these three approaches, radiation is delivered during a different point in the respiratory cycle. Intuitively, one might assume that gating under DIBH would result in a smaller mass of lung tissue being irradiated because the lung is fully expanded, and hence, its density is lower. The purpose of this study was to compare the masses of lung tissue irradiated during radiation treatments delivered at the three points in the respiratory cycle when gating is most likely to occur: (1) DIBH near 60% vital capacity (VC), (2) breath‐hold near 100% tidal volume, such as that used in ABC, and (3) breath‐hold near end expiration (0% tidal volume), such as that experienced during passive machine gating. The hypothesis was that the amount of lung tissue spared during respiratory‐gated treatments would increase as the amount of air in the lung increased. A second goal of this study is to correlate the amount of lung irradiated to various tumor characteristics such as volume of the GTV, proximity to the chest wall, location of the tumor, results of pulmonary function testing (PFT), and changes in lung density.

## II. MATERIALS AND METHODS

### Patient data acquisition

Ten patients diagnosed with primary lung carcinoma consented to participate in this study (The University of Texas M. D. Anderson Cancer Center Protocol ID00‐202 [C.W.S., Principal Investigator]). Patients with a pathologically determined diagnosis of lung cancer who had not undergone chemotherapy or surgery for their disease were enrolled in this study. Patients were immobilized using an extended wing‐board with a T‐bar hand‐grip in conjunction with an immobilization device (VacLoc™; Med‐Tech, Orange City, IA), as is our standard clinical practice, and computed tomography (CT) image data sets were obtained on a single‐slice helical scanner (PQ5000; Philips Medical Systems, Cleveland, OH). Initially, a CT image data set of the patient's entire lung during free breathing was acquired. The scan parameters were 3‐mm slice thickness in helical mode, 120 kVp, 175 mA, and a pitch of 1.5. Gated data sets were acquired at several points during the respiratory cycle using an assisted breath‐hold technique based on an occlusion spirometry system (Vmax 22; SensorMedics, Yorba Linda, CA). The spirometer was calibrated for each patient according to the manufacturer's recommendations. Data sets were typically obtained at full inspiration, corresponding to 100% tidal volume (100% TV), full expiration, corresponding to 0% tidal volume (0% TV), and, when possible, at a point corresponding to a deep‐inspiration breath‐hold, typically at 60% vital capacity (60% VC), although for some patients the deep‐inspiration breath‐hold occurred at other points in the respiratory cycle. In subsequent discussion, this deep‐inspiration point will be referred to as DI. Breath‐hold CT image data sets were acquired with a slice spacing of 0.5 cm. Gated data sets for six patients were limited to the region immediately around the tumor that could be acquired with a single breath‐hold. Subsequently, a technique for merging several gated data sets was developed that allowed a larger region to be scanned during several breath‐holds. This technique was used on the remaining four patients.

### Treatment planning

Consistency in outlining gross tumor volumes (GTV) is necessary in this study, because errors in the outlines of GTVs can lead to errors in the determination of irradiated lung volumes. In order to maximize the consistency of the outlining of the GTV in all data sets, maximum and minimum CT numbers were determined for the physician‐outlined GTV and used as thresholds to generate autocontoured GTVs. This approach was possible in seven out of the ten patient cases. In the remaining three cases, it was not possible to establish accurate minimum and maximum CT values because the tumors extended into the mediastinum, and differences between the CT values for the tumor and the mediastinum were too small. The radiation oncologist (C. W. S.) reviewed each autocontoured image. In two cases, the radiation oncologist recommended expanding the GTV by a small margin to encompass the GTV more accurately.

The CTV was determined by expanding the GTV by 0.8 cm in all three dimensions to account for microscopic disease.^(11)^ The planning target volume (PTV) for the free‐breathing plan was determined by uniformly expanding the CTV by 1.8 cm. This margin was designed to account for both setup uncertainty (measured to be 0.8 cm using the referenced setup technique) and organ motion (assumed to be 1.0 cm on the basis of current standard of practice). For the purposes of this study, the PTVs for gated treatment only included margins for setup uncertainty and were determined by expanding the CTV by 0.8 cm in all directions with no margin to account for organ motion.

To determine the differences in the amount of irradiated lung tissue between gating points, the beam geometries and treatment portals from the clinically used free‐breathing (FB) plan were copied to each gated CT data set. These treatment plans were designated as FB – 0 TV, FB – 100 TV, and FB – DI. The isocenter of all four beams was moved from the center of the free‐breathing PTV to the center of the PTV for the particular data set. A second set of treatment plans was developed for the gated studies using a 1‐cm expansion of each gated PTV to define the treatment portal. The second set of plans was completed to compare the amount of lung tissue irradiated at various points in the respiratory cycle using margins that simulate those used with gating. The images and contours were transferred to another treatment planning system^(12)^ in which the beams were set up and doses were calculated. For the six patients for whom the gated CT image data set was acquired under a single breath hold and did not include the entire lung, it was necessary to extend the CT image data set to ensure that the entire treatment portal fell within the patient image. For these cases, we extended the data set by replicating the first and last axial images for a distance of 10 cm.

### Analysis of treatment plans

In order to compare treatment plans, both dose‐volume histograms (DVH) and dose‐mass histograms (DMH) of the ipsilateral lung were calculated.^(13)^ Although DVHs have traditionally been used to evaluate effects of radiation, DMHs may be more useful in this study for several reasons. First, for approximately half of the patients in this series, we were unable to obtain breath‐hold CT image data sets comprising the entire lung volumes. Typically, DVHs display the fractional volume of lung irradiated vs dose. However, because the total volume of lung is not known, the fractional lung volume cannot be determined. Second, in comparing treatment plans based on CT data sets acquired during various phases in the respiratory cycles, we encountered varying densities of lung. Typical lung densities under deep inspiration are significantly less than densities under full inspiration, which are less than densities under full expiration. DMHs account for these differences in lung density while DVHs do not. Finally, radiation effects are based on the number of cells of lung tissue irradiated. Because the volume of lung changes during respiration, the mass of lung tissue is likely to be a better representation of the actual number of lung cells damaged by radiation than the volume of lung tissue. Thus while the total volume of irradiated lung, which can be obtained from partial‐volume CT data sets, is not necessarily a good indicator of potential lung toxicity, the total mass of irradiated lung, which can be obtained from DMHs, can serve as an indicator. In this study DMHs were computed using the method described by Forster et al. ^(13)^ In this method, the volume of each CT voxel is weighted by its local density, obtained from the CT to density conversion table in the treatment planning system.

DMHs extracted from dose distributions calculated using the treatment portals based on free‐breathing PTVs were compared to DMHs extracted from dose distributions calculated using the treatment portals based on the breath‐hold PTVs. DMHs extracted from dose distributions calculated from breath‐hold image data sets at the different points in the respiratory cycle were compared to each other to determine whether the masses of lung tissue irradiated at different points in the respiratory cycle were significantly different from each other. These comparisons were repeated for all patients in the study. The masses of lung tissue receiving at least 20 Gy, denoted as M20, were used to assess the potential toxicity of the treatment plans. Our clinical experience has been to use the volume of lung tissue receiving at least 20 Gy (V20) for this purpose.^(14)^ However, in the present study we compared results in an environment in which the density of the lung tissue changed because of respiration.

M20 values were determined from the plans using the gated PTV portal at each gating point. Ratios of these M20 values to the M20 value from the plan using the free‐breathing portal were calculated. These M20 ratios determined the reduction in mass of irradiated lung tissue effected by gating. It should be noted that this approach only takes into account static M20 values. This approach is very reasonable for the gated studies, but the mass of lung tissue irradiated during free‐breathing is probably underestimated because the mass of lung tissue moving into and out of the fields is not taken into account.

Ratios of M20 at deep inspiration to M20 at 0% TV and ratios of M20 at 100% TV to M20 at 0% TV were evaluated to determine any potential advantages of gating at specific gating points. These M20 ratios were compared with GTV volumes, tumor location (proximity to the chest wall), tumor location (apex, middle, or base of the lung), pulmonary function test data, and percent change in the lung density to be evaluated for any disease characteristics that might predict a greater need for gating. The correlations were quantified using linear regression and unpaired t‐tests.

## III. RESULTS

The M20 ratios for the plans using portals based on PTVs versus the ratios for the plans using the free‐breathing plan portals were extracted from the DMHs calculated at each gating point to illustrate the reduction in irradiated lung mass effected by gating. These results are presented in Table I. The M20 ratios in this table show that in every case, the mass of lung tissue irradiated using a gated technique would be lower than it would in ungated cases. The M20 ratios for the plans based on the free‐breathing portal are included in the data because they support our hypothesis that a smaller mass of lung issue was treated at a higher inspiration level. The mean reduction in the M20 of the 10 patients studied was 19% (SD±0.11), and the range was 0.57 –0.97. This is comparable to the mean reduction in V20, which was also 19%. Reductions in the amount of irradiated lung were the consequence of the reduction in margins.

**Table I:** M20 ratios for ipsilateral lung for gated PTV portals vs free‐breathing portal indicating potential reduction in mass of ipsilateral lung irradiated (DI – deep inspiration; TV – tidal volume)

Patient #	DI	100% TV	0% TV	Mean
01	0.92	0.86	0.80	0.86
02	0.71	0.82	0.88	0.80
03	0.78	0.82	0.77	0.79
04	0.57	0.60	0.62	0.60
05	0.83	0.83	0.87	0.84
06	0.77	0.74	0.71	0.74
07	0.84	0.87	0.85	0.85
08	0.97	0.96	0.97	0.97
09		0.95	0.95	0.95
10		0.79	0.67	0.73
Mean	0.80	0.82	0.81	

**Figure 1:**
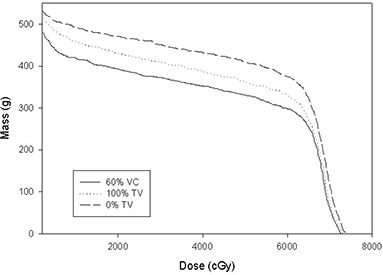
Dose‐mass histograms for ipsilateral lung for Patient 04 comparing deep inspiration (60% vital capacity), 100% tidal volume and 0% tidal volume using the free‐breathing portal.

**Figure 2:**
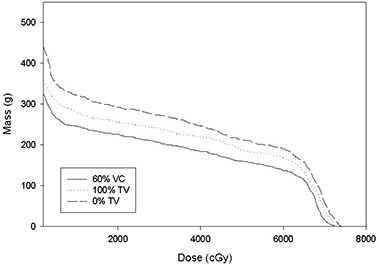
Dose‐mass histograms for ipsilateral lung for Patient 04 comparing deep inspiration (60% vital capacity), 100% tidal volume and 0% tidal volume using the portal based on the gated planning target volumes.

Fig. 1 provides an example of the DMH for a patient's left lung using the free‐breathing plan. M20 was less at deep inspiration than at the other two gating points and less at 100% tidal volume than at 0% tidal volume. This is typical of most of the patient data. Fig. 2 illustrates the DMH for the same patient's left lung using the portal based on the gated PTVs. M20 was again less at deep inspiration than at the other two gating points and less at 100% tidal volume than at 0% tidal volume. This is also typical of most of the patient data in this study.

In order to assess the amount of lung tissue spared as a function of the gating point, the ratios of M20 values for deep inspiration versus 0% TV and 100% TV versus 0% TV were calculated. The M20 ratios demonstrate that not much more tissue was spared gating at 100% TV versus 0% TV; the average M20 ratio for the plan using the free‐breathing portal was 0.95 while the average M20 ratio for the plan using the PTV‐based portal was 0.96 (Table II). More lung tissue was spared gating at the deep inspiration level (deep inspiration versus 0% TV); the average M20 ratio for the plan using the free‐breathing portal was 0.90, while the average M20 ratio for the plan using the PTV‐based portal was 0.89 (Table III). The data from the plans using the portal based on the PTV seem to be the most important in evaluating clinical significance because the margins used in the plans are those that would be used for clinical purposes.

**Table II:** Comparison of mass of irradiated ipsilateral lung receiving 20 Gy for 100% tidal volume vs 0% tidal volume for all patients.

Patient #	M20(100% TV)/M20(0% TV) based on free‐breathing portal	M20(100% TV)/M20(0% TV) based on PTV	Mean
01	1.00	1.08	1.04
02	0.97	0.90	0.94
03	0.95	1.01	0.98
04	0.91	0.87	0.89
05	0.99	0.94	0.99
06	0.89	0.93	0.91
07	0.95	0.97	0.96
08	1.01	1.00	1.00
09	0.97	0.97	0.97
10	0.89	0.95	0.92
Mean	0.95	0.96	

**Table III:** Comparison of mass of irradiated ipsilateral lung receiving 20 Gy for deep inspiration vs 0% tidal volume for all patients.

Patient #	M20(DI)/M20(0% TV) based on free‐breathing portal	M20(DI)/M20(0% TV) based on PTV	Mean
01	0.93	1.07	1.00
02	0.94	0.76	0.85
03	0.97	0.99	0.98
04	0.84	0.77	0.81
05	0.88	0.84	0.86
06	0.84	0.92	0.88
07	0.80	0.79	0.80
08	0.96	0.96	0.96
Mean	0.90	0.89	

The masses of lung tissue irradiated were reduced at all points in the respiratory cycles, indicating that normal tissue was spared by gating, irrespective of the point at which gating was effected. Based on the M20 ratios, we find that the reduced portal allowed by respiratory gating spared as much as 43% of normal lung tissue.

Patients 08 and 09 experienced little benefit at any gating point indicating that individualization of gating technique is required for optimal effectiveness. Note that patients 09 and 10 underwent only 2 CT scans: one at 0% TV and one at 100% TV. For these patients, the scans at 100% TV corresponded to approximately deep inspiration. The data are identified in the tables at 100% TV for comparison purposes.

Respiratory gating is a time‐consuming, labor‐intensive, and costly process. It would be helpful if easily obtainable patient or tumor characteristics could predict whether a patient would experience any significant benefit from gating. Therefore, the M20 ratios were correlated with several characteristics including the average volume of the GTV, the proximity of the tumor to the chest wall, the tumor location (apex, middle, or base), pulmonary function test data, and percent change in lung density. Table IV shows the patient characteristics and the corresponding p‐value for the correlation to the M20 ratios. Only one patient in the comparison had a tumor in the middle of the lung, so the t‐test could not be performed for this correlation. The data in Table IV show that there is likely no correlation between lung tissue sparing and these patient characteristics. Therefore, none of these patient characteristics seem to be valid predictors of any benefits of gating in this patient sample. These data are consistent with the findings of Stevens et al., ^(15)^ who found no correlation between tumor location, tumor size, or PFT values and magnitude of respiratory‐induced tumor motion.

**Table IV:** Correlation of patient characteristics with M20 ratios as indicated by p‐values

	DI/0% TV		100% TV/0% TV	
Patient characteristics	Free‐breathing portal	Gated PTV portal	Free‐breathing portal	Gated PTV portal
GTV Volumes	0.97	0.36	0.85	0.65
Proximity to chest wall			0.57	0.51
Tumor location	0.82	0.97	0.87	0.49
% of predicted VC	0.03	0.26	0.34	0.72
VC	0.81	0.995	0.96	0.51
Volume of air	0.05	0.14	0.05	0.14
% change in lung				
density	0.11	0.45	0.91	0.50

## IV. DISCUSSION

Gating the delivery of radiation treatment to a pre‐determined point in the respiratory cycle has been identified as a potential method for reducing the size of the ITV and decreasing the amount of uninvolved lung tissue placed at risk from irradiation. In this preliminary study, we assessed the potential for sparing of lung tissue during gating by comparing the masses of lung tissue receiving at least 20 Gy, an indicator of potential lung toxicity.

In an attempt to identify predictors for assessing potential benefit of gating, the M20 ratios were compared to the average of the gated GTV volumes, tumor proximity to the chest wall, tumor location (apex, middle, or base), data obtained from pulmonary function tests, and percent change in the lung density. Our analysis of the correlation between M20 ratios and disease characteristics did not reveal any means to determine which patients would benefit the most from respiratory gating. Consequently, the only indicator that we have been able to determine prior to treatment is whether the patient can withstand a breath‐hold technique.

Several sources of error may have been introduced into the assessment of DMHs. Two sources of uncertainty may occur in the contouring of the GTVs. The GTV contours for the same patient on all gated scans were not exactly the same. Changes in the lung density associated with respiration may cause different placement of the boundary separating GTV from lung. The change in the GTV affects the size of the treatment portal because the PTV is delineated from the expansions of the GTV. If the size of the treatment portal were slightly different because of these small differences in GTV, then a slightly different amount of lung tissue would be irradiated. A second source of GTV uncertainty may be in the partial volume effect associated with the 5‐mm thickness of the CT slices acquired for the gated scans.

Another source of uncertainty lies in the small differences between M20 values at different points in the respiratory cycle. Because tumor is not considered normal lung tissue, the amount of tissue defined as lung is the lung contour minus the GTV. Consequently, the small differences in GTV volumes between different data sets may change the amount of tissue being defined as normal lung tissue. This difference may account for the small (~1%) differences in the mass of total lung tissue irradiated.

Yet another source of uncertainty is the magnitude of the margin used to expand the CTV to form the PTV. In the analysis, we expanded the CTV by 1.8 cm for free‐breathing treatment and 0.8 cm for gated treatment. This expansion assumes no residual motion during gating. In present gating practice, some residual motion exists, and properly should be included in the analysis. However, newer techniques that combine gating with feedback‐guided breath‐hold (FGBH) allow significant reduction in residual motion under gating, and make the 0.8 cm margin realistic.

Other sources of uncertainty include (1) systematic errors in the dose calculation algorithms in the treatment planning system, (2) uncertainties in the breath‐hold points determined by the spirometer system, and (3) determination of the local density from the CT number to density conversion table. These errors are believed to have a negligible effect on uncertainties in lung tissue.

## V. CONCLUSIONS

In this preliminary study, we found no predictors of whether gating on expiration or gating on inspiration spares more normal lung tissue during irradiation. The only issue that can be resolved before the simulation is whether or not a patient can withstand a breath‐hold.

Respiratory gating decreased the amount of lung tissue irradiated for every patient in this study at every point in the respiratory cycle, but there did not appear to be significant differences in the amount of lung tissue irradiated at 100% TV versus that irradiated at 0% TV. This indicates that for the patient who cannot withstand a DIBH, passive gating at 0% TV would benefit them as much as would at 100% TV. The primary advantage of gating at 0% TV is that the procedure appears to be easier on the patient and requires less personnel involvement. Passive gating at 0% TV allows the patient to breathe normally. We have found that commercially‐available devices that support passive gating (e.g., the RPM™, Varian Medical Systems, Inc., Palo Alto CA) are easier to use than occlusion spirometers; however, the point at which gating occurs seems to be more reproducible in spirometry‐based systems than using passive gating techniques.

## ACKNOWLEDGEMENTS

This research was supported in part by a sponsored research agreement with Varian Medical Systems, Inc., Palo Alto, CA.
